# Dietary heterocyclic aromatic amine intake and cancer risk: epidemiological evidence from Japanese studies

**DOI:** 10.1186/s41021-021-00202-5

**Published:** 2021-07-27

**Authors:** Motoki Iwasaki, Shoichiro Tsugane

**Affiliations:** 1 Division of Epidemiology, Center for Public Health Sciences, National Cancer Center, Tokyo, Japan; 2 National Institute of Health and Nutrition, National Institutes of Biomedical Innovation, Health and Nutrition, Tokyo, Japan

**Keywords:** Heterocyclic aromatic amines, Dietary intake, Food frequency questionnaire, Validity, Colorectal cancer, Colorectal adenoma, Prostate cancer, Epidemiological study

## Abstract

Heterocyclic aromatic amines (HAAs), which are formed from the reaction of creatine or creatinine, amino acids, and sugars in meat and fish cooked at high temperatures, have been shown to be mutagenic in bacterial assays and carcinogenic in animal models. Following advances in the dietary assessment of HAA intake in epidemiological studies - including development of a validated meat-cooking module and a specialized food composition database - a number of epidemiological studies have specifically examined the association of HAA intake and cancer risk, most of which were conducted in Western countries. Given that dietary habits and cooking methods differ across countries, however, epidemiological investigation of dietary HAA intake requires a population-specific assessment method. Here, we developed a practical method for assessing dietary HAA intake among Japanese using a food frequency questionnaire (FFQ) and evaluated its validity for use in epidemiological studies by comparison with 2-amino-1-methyl-6-phenylimidazo [4,5-*b*] pyridine (PhIP) levels in human hair. The Japan Public Health Center-based Prospective Study reported that daily intake of HAAs among Japanese was relatively low, and that more than 50% of total intake in mainland Japan was derived from fish. Only four case-control studies in Japan have been reported so far, for colorectal, stomach and prostate cancer, and colorectal adenoma. A statistically significant positive association was found between 2-amino-3,4-dimethylimidazo [4,5-f] quinoline (MeIQ) and the risk of colorectal adenoma and between individual and total HAAs and the risk of prostate cancer. In contrast, no association was observed for colorectal or stomach cancer, or for colorectal adenoma among men. We also found that the limited and inconsistent findings among epidemiological studies are due to the difficulty in assessing exposure levels of HAAs. In addition to further evidence from prospective cohort studies in Japanese based on dietary HAA intake estimated by FFQs, studies using other methods to assess HAA exposure, such as biomarkers, are highly anticipated.

## Background

Heterocyclic aromatic amines (HAAs), which are formed by the reaction of creatine or creatinine, amino acids and sugars in meat and fish to cooking at high temperatures, have been shown to be mutagenic in bacterial assays and carcinogenic in animal models [1–3]. They act through the formation of DNA adducts after metabolic activation via N-oxidation by cytochrome P-450 1A2 (CYP1A2), followed by O-acetylation by *N*-acetyltransferase 1 (NAT1) and *N*-acetyltransferase 2 (NAT2) [3, 4]. Although over 25 mutagenic HAAs have been identified, the most abundant formed in meats are 2-amino-1-methyl-6-phenylimidazo [4,5-*b*] pyridine (PhIP), 2-amino-3,8-dimethylimidazo [4,5-*f*] quinoxaline (MeIQx), and 2-amino-3,4,8-trimethylimidazo [4,5-*f*] quinoxaline (4,8-DiMeIQx) [4]. Monographs published in 1993 by the International Agency for Research on Cancer (IARC) classified PhIP, 2-amino-3,4-dimethylimidazo [4,5-*f*] quinoline (MeIQ) and MelQx as ‘possible human carcinogens’ (Group 2B) because of a lack of evidence from epidemiological studies [5]. Since then, advances in the dietary assessment of HAA intake in epidemiological studies - including development of a validated meat-cooking module and a specialized food composition database [6] - led to a number of epidemiological studies that specifically examined the association of HAA intake and cancer risk, mainly from Western countries [7–13].

The formation of HAAs increases with the temperature and duration of cooking and varies with the type of meat and cooking method. The highest levels are produced by pan-frying, barbecuing, and grilling [4, 14]. These cooking methods are not equivalent across countries and populations, however. Japanese generally consume more fish than animal meat, and the chopped and stir-fried method of meat preparation is favored as much as the grilled cooking method [15]. Thus, foods contributing to HAA intake in Japan probably differ from those consumed in Western countries, which in turn indicates the necessity of population-specific methods to assess dietary HAA intake in epidemiological studies. In this article, we review Japanese studies from the development of dietary assessment to its application in epidemiological studies.

### Development of dietary assessment method

We developed a practical method for assessing dietary HAA intake using a food frequency questionnaire (FFQ) used for the Japan Public Health Center-based Prospective Study (JPHC study) [16]. First, a composition table of HAAs was developed using fish and meat items selected from the food list in the FFQ. Among 19 fish items, we selected six which are usually grilled. We then disaggregated the two of these six which are aggregate items, and finally measured HAA content in eight fish species, namely atka mackerel (“hokke”; salted) for salted fish, horse mackerel (“aji”; salted and semi-dried split) for semi-dried split fish, salmon (“sake”; raw), horse mackerel (“aji”; raw), sardine (“iwashi”; raw), Pacific saury (“sanma”; raw), mackerel (“saba”; raw), and eel (“unagi”; seasoned) [17]. Among three meat items (beef, pork and chicken) with five different types of cooking preparation (pan-fried and stewed for beef; stir-fried, deep-fried and stewed for pork; grilled and deep-fried for chicken), HAA content was measured for pan-fried beef, stir-fried pork, and grilled chicken [17]. We then used the measured values for each food item to develop an HAA composition table to estimate dietary HAA intake from the FFQ (Table 1). For processed meat items, the composition table included published values for bacon.

**Table 1:** Composition table of heterocyclic aromatic amines for estimating dietary intake from the food frequency questionnaire (ng/100 g)^a^

	PhIP	MeIQx	MelQ	4,8-DiMeIQx	7,8-DiMeIQx	IQ	Trp-P-1
Fish							
Salted fish, flesh	37	8	17	0	0	0	0
Salted fish, skin	700	59	9	9	446	0	29
Semi-dried fish, flesh	52	0	7	0	0	0	0
Semi-dried fish, skin	368	42	26	55	95	0	28
Salmon, flesh	29	10	18	0	0	0	0
Salmon, skin	593	59	0	0	414	0	29
Horse mackerel and Sardine, flesh	8	9	0	0	3	0	0
Horse mackerel and Sardine, skin	147	62	16	69	0	7	11
Pacific saury and Mackerel, flesh	28	10	3	0	0	0	0
Pacific saury and Mackerel, skin	160	54	40	60	37	10	0
Eel, flesh	15	0	22	0	0	0	0
Eel, skin	11	0	0	0	0	0	0
Meat							
Pan-fried beef, well-done	59	17	11	0	0	0	0
Stir-fried pork	86	20	9	0	0	0	0
Grilled chicken	29	8	14	0	0	0	53
Bacon	33	13	0	3	0	0	0

*4,8-DiMeIQx* 2-amino-3,4,8-trimethylimidazo [4,5-*f*]quinoxaline, *7,8-DiMeIQx* 2-amino-3,7,8-trimethylimidazo [4,5-*f*]quinoxaline, *IQ* 2-amino-3-methylimidazo [4,5-*f*]quinoline, *MeIQx* 2-amino-3,8-dimethylimidazo [4,5-*f*]quinoxaline, *MeIQ* 2-amino-3,4-dimethylimidazo [4,5-*f*]quinoline, *PhIP* 2-amino-1-methyl-6-phenylimidazo [4,5-*b*]pyridine, *Trp-P-1* 3-amino-1,4-dimethyl-5H-pyrido [4,3-*b*]indole

^a^Data are from Kobayashi et al. [16]

Second, we established a calculation method for dietary HAA intake using the FFQ and the composition table of HAAs [16]. The FFQ consists of food items with nine frequency categories and standard portions/units, and asks about the usual consumption of listed foods during the previous year. Daily food intake is calculated by multiplying frequency by standard portion and relative size for each food item. The FFQ includes additional questions about grilled skin consumption using the five quantity categories of almost none, one-third, half, two-thirds, to almost all, and preferred doneness levels (very well-done, well-done, medium, medium rare, and rare) for pan-fried and grilled beef. HAA intake from fish consumption was estimated based on the proportion of grilled to total fish consumption, the rate of grilled skin consumption, the ratio of skin to flesh, and data on HAA content in the skin and flesh. HAA intake from meat consumption was estimated based on preferred doneness levels and data on HAA content.

### Validity of dietary assessment method

Evaluating the validity of the FFQ in estimating dietary HAA intake requires a reference method which reflects long-term exposure to dietary HAA intake. A promising biomarker of PhIP intake is PhIP accumulation in human hair [18]. A feeding study among non-smokers using ground beef cooked to two different levels of doneness showed a strong dose-dependent increase in hair PhIP levels [19]. Therefore, we first established an analytical method for PhIP in human hair using liquid chromatography-electrospray ionization/tandem mass spectrometry (LC-ESI/MS/MS) [20]. We then verified the quantitative performance of the analytical method using rat fur samples with a controlled level of exposure to PhIP by investigating the dose–response relationship between three exposure levels (control, 20 ppm, and 200 ppm) in fur collected at four different times. A positive dose–response relationship was observed between dosage and PhIP accumulation in rat fur regardless of the time of fur collection. To clarify whether the PhIP levels in rat fur samples represented the exposure level to PhIP in target tissue, we also examined PhIP-DNA adduct levels in the rat colon, a target organ for putative HAA-associated cancer, and observed a similar pattern of PhIP levels as those detected in rat fur. These findings reconfirmed that hair samples can be used as a surrogate tissue for evaluation of exposure levels [18, 21].

**Fig. 1:**
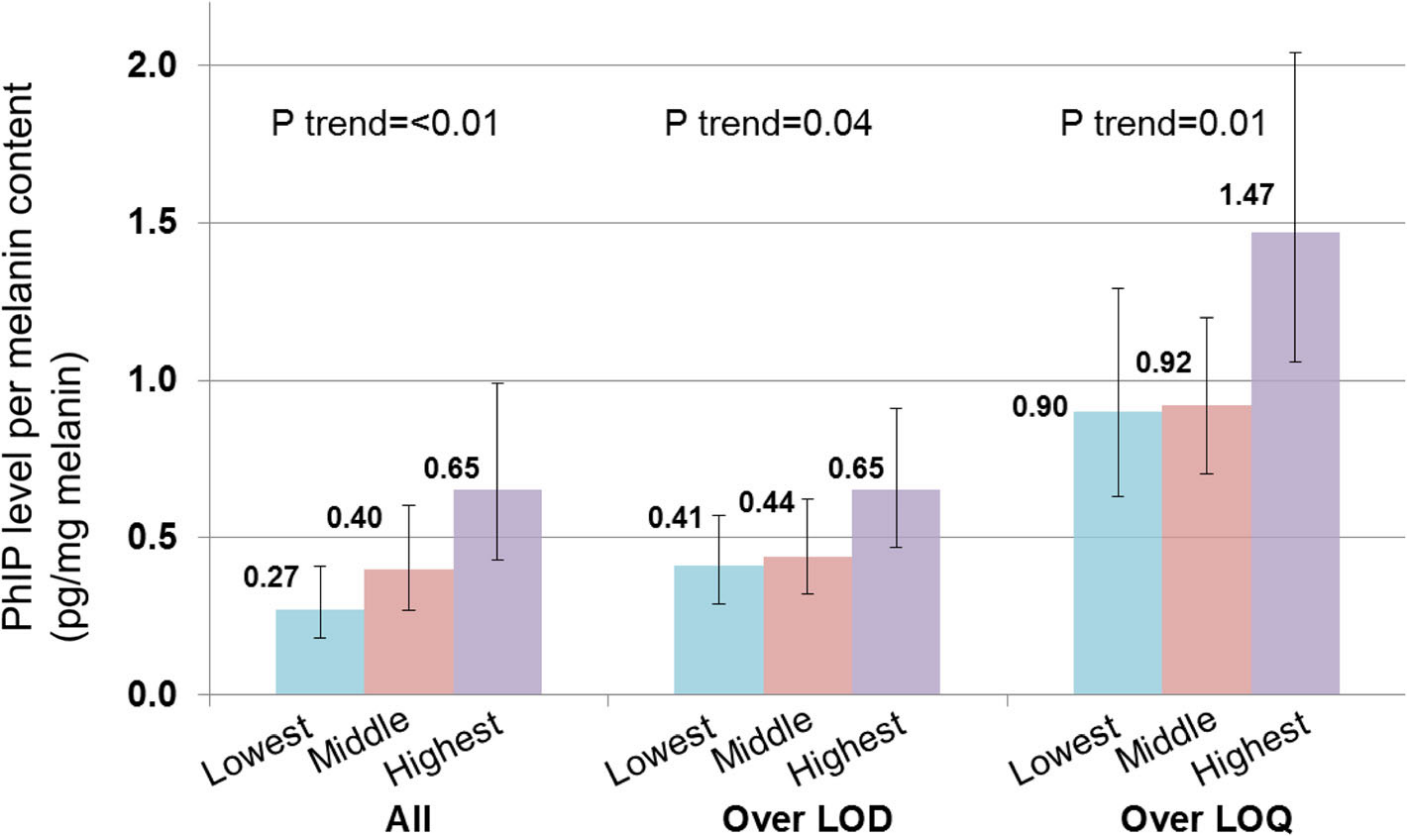
Adjusted geometric mean and 95% confidence interval of PhIP level per melanin content in human hair (pg/mg melanin) according to tertile category of energy-adjusted PhIP intake. Data are from Iwasaki et al. [20] LOD: limit of detection; LOQ: limit of quantification; PhIP: 2-amino-1-methyl-6-phenylimidazo [4,5-*b*]pyridine.

We conducted a validation study among examinees of a cancer screening program provided by the National Cancer Center, Japan [20]. Among 65 examinees, measured PhIP levels were over the limit of detection (LOD) in 57 subjects and over the limit of quantification (LOQ) in 24. Dietary intake of the following seven HAAs was calculated: PhIP, MeIQx, MeIQ, 4,8-DiMeIQx, 2-amino-3,7,8-trimethylimidazo [4,5-f] quinoxaline (7,8-DiMeIQx), 2-amino-3-methylimidazo [4,5-f] quinoline (IQ) and 3-amino-1,4-dimethyl-5H-pyrido [4,3-b] indole (Trp-P-1). Total HAA intake was defined as the sum of the seven HAAs above, and the three HAAs with the highest intake (PhIP, MeIQx, and MeIQ) were used for the following analyses. We compared PhIP levels in human hair with dietary HAA intake estimated from the FFQ. Spearman rank correlation coefficients between energy-adjusted HAA intake from the FFQ and PhIP level per melanin content in hair showed a significant positive correlation coefficient range of 0.32 to 0.36 among all participants regardless of type of HAA (Table 2). However, somewhat lower correlation coefficients were observed among participants with over-LOD or -LOQ values, albeit that these were not statistically significant. In addition, adjusted geometric mean levels of PhIP level per melanin content in hair according to tertile category of energy-adjusted PhIP intake were calculated using a multivariable linear regression model with adjustment for sex, age, and body mass index. PhIP levels were significantly increased with a higher category of energy-adjusted PhIP intake for three groups (all participants, and participants with over-LOD or -LOQ values) (Fig. 1). These findings suggest that our FFQ for middle-aged or older Japanese is reasonably valid for the assessment of HAA intake, which in turn allows its use in future epidemiological studies of the association between HAA intake and cancer risk by ranking individuals by dietary intake of HAAs.

**Table 2:** Spearman rank correlation coefficients and 95% confidence intervals of energy-adjusted HAA intake and PhIP level per melanin content in hair^a^

	All subjects		Subjects with over LOD values		Subjects with over LOQ values	
	CC	95% CI	CC	95% CI	CC	95% CI
PhIP intake	0.35	(0.11, 0.54)	0.21	(−0.05, 0.45)	0.34	(−0.07, 0.66)
MeIQ intake	0.36	(0.13, 0.56)	0.24	(−0.02, 0.47)	0.20	(−0.22, 0.56)
MeIQx intake	0.32	(0.08, 0.52)	0.24	(−0.02, 0.47)	0.17	(−0.25, 0.53)
Total HAA intake	0.34	(0.11, 0.54)	0.22	(−0.05, 0.45)	0.23	(−0.19, 0.58)

*CC* correlation coefficient, *CI* confidence interval, *HAA* heterocyclic aromatic amine, *LOD* limit of detection, *LOQ* limit of quantification, *MeIQ* 2-amino-3,4-dimethylimidazo [4,5-*f*]quinoline, *MeIQx* 2-amino-3,8-dimethylimidazo [4,5-*f*]quinoxaline, *PhIP* 2-amino-1-methyl-6-phenylimidazo [4,5-*b*]pyridine

^a^Data are from Iwasaki et al. [20]

### Dietary HAAs intake among Japanese

We calculated dietary HAA intake for 39,035 men and women from four public health center (PHC) areas (Ninohe PHC area in Iwate, Yokote PHC area in Akita, Saku PHC area in Nagano, and Chubu PHC area in Okinawa) in the JPHC study [16]. Total HAA intake was defined as the sum of intake of PhIP, MeIQx, MeIQ, 4,8-DiMeIQx, 7,8-DiMeIQx, and Trp-P-1, based on the FFQ in the 5-year follow-up survey. Because different dietary habits were recognized between the mainland areas and Okinawa Island [15], their results are presented separately. Mean daily intake levels of total HAAs (ng/kg per day) for participants in the mainland areas (Iwate, Akita, and Nagano) were 1.06 (inter quartile range 0.50–1.35) in men and 1.10 (inter quartile range 0.53–1.40) in women. Those for participants in Okinawa Island were lower than those in the mainland, at 0.83 (inter quartile range 0.33–1.04) in men and 0.92 (inter quartile range 0.33–1.18) in women. This difference might be explained by a difference in cooking methods, since grilling was not the main method for preparing foods in Okinawa. Meanwhile, daily intake levels among Japanese might be lower than those in Western countries (e.g. 9 ng/kg per day in the US population), albeit that differences in study design and assessment method hindered comparison [4, 22].

The contribution of fish or meat to HAA intake is shown in Table 3. More than 50% of total HAA intake in the mainland participants was derived from fish, whereas more than 50% in Okinawa was from pork, which might reflect differences in dietary habits. Consumption of meat was higher in Okinawa than on the mainland and pork was the preferred meat. In a US study [23], chicken was the largest source of HAAs among different meat types. In contrast, chicken contributed only 10% of HAA intake in Japan.

**Table 3:** Intake of total heterocyclic aromatic amines from fish and meat (ng per day (%))^a^

	Mainland				Okinawa			
	Men		Women		Men		Women	
	ng per day	(%)	ng per day	(%)	ng per day	(%)	ng per day	(%)
Fish	36.21	(55.0)	31.86	(54.5)	6.62	(12.5)	5.45	(10.8)
Meat								
Beef	8.22	(12.5)	6.42	(11.0)	11.29	(21.4)	9.83	(19.4)
Pork	13.81	(21.0)	13.73	(23.5)	29.37	(55.6)	30.47	(60.3)
Chicken	7.22	(11.0)	6.08	(10.4)	4.83	(9.1)	4.12	(8.2)
Processed	0.33	(0.5)	0.38	(0.7)	0.70	(1.3)	0.69	(1.4)

^a^Data are from Kobayashi et al. [16]

### Epidemiological evidence for cancer risk

To date, only four epidemiological studies have examined associations between HAA intake and cancer risk among Japanese. Their main findings are summarized in Table 4. Colorectal cancer and adenoma, well-known precursor lesions of cancer, have been most extensively studied [12, 13]. We failed to detect significant associations between dietary HAA intake and the risk of colorectal cancer based on a hospital-based case-control study in Nagano, although the odds ratio (OR) (95% confidence interval [CI]) in the highest tertile category of MeIQx was 1.98 (0.42–9.32) [24]. A recent meta-analysis of colorectal cancer showed statistically significant associations for MeIQx and DiMeIQx [12]. However, only three cohort studies have investigated the association between HAA intake and the risk of colorectal cancer [7, 9, 11]. One of these found positive associations of MeIQx and DiMeIQx with the risk of colon cancer [7] whereas the other two observed no associations for individual or total HAAs [9, 11]. Further evidence from prospective cohort studies among Japanese is needed.

**Table 4:** Summary of epidemiological studies of associations between HAA intake and cancer risk among Japanese

Reference	Study period	Outcome	Design	Study subjects			Exposure	Category	Odds ratios	95% confidence intervals	P for trend	Confounding factors
				Definition	Age range	Number of subjects						
Kobayashi et al. [24]	1998–2002	Colorectal cancer	Hospital-based case-control	Cases: histopathologically confirmed, Controls: medical check-up examinees	21–76 years	117 cases and 238 controls	PhIP	Tertile 1	1		0.777	Matched (1:2) for sex, age and area of residence. Adjusted by conditional logistic regression for smoking status, alcohol intake, family history of colorectal cancer, body mass index, JA membership, and intake of vegetables, meat, fish, and dietary fiber.
								Tertile 2	1.69	(0.52–5.56)		
								Tertile 3	1.32	(0.27–6.48)		
							MeIQx	Tertile 1	1		0.397	
								Tertile 2	1.67	(0.51–5.41)		
								Tertile 3	1.98	(0.42–9.32)		
							MeIQ	Tertile 1	1		0.793	
								Tertile 2	1.09	(0.28–4.25)		
								Tertile 3	1.23	(0.23–6.64)		
							Total HAAs	Tertile 1	1		0.903	
								Tertile 2	1.77	(0.55–5.72)		
								Tertile 3	0.99	(0.21–4.81)		
Kobayashi et al. [25]	1998–2002	Stomach cancer	Hospital-based case-control	Cases: histopathologically confirmed, Controls: medical check-up examinees	21–76 years	149 cases and 296 controls	PhIP	Tertile 1	1		0.556	Matched (1:2) for sex, age and area of residence. Adjusted by conditional logistic regression for *H. pylori* status, smoking status, alcohol intake, family history of stomach cancer, body mass index, total vegetable intake, meat intake, fish intake, salt intake, and JA membership.
								Tertile 2	1.75	(0.77–3.99)		
								Tertile 3	1.33	(0.44–4.02)		
							MeIQx	Tertile 1	1		0.248	
								Tertile 2	1.06	(0.52–2.14)		
								Tertile 3	0.81	(0.36–1.82)		
							MeIQ	Tertile 1	1		0.922	
								Tertile 2	1.23	(0.52–2.89)		
								Tertile 3	1.06	(0.36–3.12)		
							Total HAAs	Tertile 1	1		0.807	
								Tertile 2	1.33	(0.59–2.98)		
								Tertile 3	1.11	(0.36–3.49)		
Budhathoki et al. [26]	2004–2005	Colorectal adenoma	Cross-sectional	Cases: diagnosed according to pit-pattern classification using magnifying colonoscopy with dye spreading, Controls: confirmed by total colonoscopy	50–79 years	498 cases and 453 controls among men	PhIP	Quartile 1	1		0.77	Adjusted for age, screening period, smoking, alcohol consumption, body mass index, physical activity, family history of colorectal cancer, and NSAID use. Further adjusted for age at menarche, menopausal status, and current use of hormones in females.
								Quartile 2	1.33	(0.91–1.93)		
								Quartile 3	1.09	(0.74–1.60)		
								Quartile 4	1.02	(0.69–1.50)		
							MeIQx	Quartile 1	1		0.97	
								Quartile 2	1.33	(0.91–1.94)		
								Quartile 3	1.26	(0.86–1.85)		
								Quartile 4	1.01	(0.68–1.50)		
							MeIQ	Quartile 1	1		0.47	
								Quartile 2	1.21	(0.84–1.76)		
								Quartile 3	0.95	(0.65–1.40)		
								Quartile 4	0.94	(0.64–1.37)		
							Total HAAs	Quartile 1	1		0.64	
								Quartile 2	1.43	(0.98–2.08)		
								Quartile 3	1.05	(0.71–1.55)		
								Quartile 4	1.02	(0.69–1.50)		
						240 cases and 244 controls among women	PhIP	Quartile 1	1		0.09	
								Quartile 2	0.74	(0.42–1.32)		
								Quartile 3	1.08	(0.62–1.88)		
								Quartile 4	1.43	(0.83–2.45)		
							MeIQx	Quartile 1	1		0.10	
								Quartile 2	1.02	(0.58–1.78)		
								Quartile 3	0.97	(0.56–1.70)		
								Quartile 4	1.58	(0.92–2.73)		
							MeIQ	Quartile 1	1		0.01	
								Quartile 2	1.42	(0.80–2.53)		
								Quartile 3	1.73	(0.97–3.08)		
								Quartile 4	2.10	(1.20–3.67)		
							Total HAAs	Quartile 1	1		0.03	
								Quartile 2	1.04	(0.58–1.86)		
								Quartile 3	1.44	(0.83–2.51)		
								Quartile 4	1.73	(0.99–3.01)		
Koda et al. [27]	2004–2006	Prostate cancer	Hospital-based case-control	Cases: histologically confirmed, Controls: medical check-up examinees	40–90 years	351 cases and 351 controls	PhIP	Tertile 1	1		< 0.001	Matched (1:1) for age. Adjusted by conditional logistic regression for alcohol intake, smoking status, body mass index, family history of prostate cancer, and total energy intake
								Tertile 2	1.06	(0.77–1.48)		
								Tertile 3	1.84	(1.35–2.50)		
							MeIQx	Tertile 1	1		< 0.001	
								Tertile 2	1.05	(0.76–1.46)		
								Tertile 3	2.25	(1.65–3.06)		
							MeIQ	Tertile 1	1		< 0.001	
								Tertile 2	1.18	(0.85–1.63)		
								Tertile 3	1.87	(1.38–2.55)		
							Trp-P-1	Tertile 1	1		< 0.001	
								Tertile 2	1.05	(0.76–1.45)		
								Tertile 3	1.92	(1.42–2.61)		
							Total HAAs	Tertile 1	1		< 0.001	
								Tertile 2	1.14	(0.83–1.58)		
								Tertile 3	1.90	(1.40–2.59)		

*HAAs* heterocyclic aromaticamines, *MeIQx* 2-amino-3,8-dimethylimidazo [4,5-*f*]quinoxaline, *MeIQ* 2-amin**o**-3,4-dimethylimidazo [4,5-*f*]quinoline, *Trp-P-1* 3-amino-1,4-dimethyl-5H-pyrido [4,3-*b*]indole, *PhIP* 2-amino-1-methyl-6-phenylimidazo [4,5-*b*]pyridine

For colorectal adenoma, we found a significant increase in risk associated with the dietary intake of MeIQ and total HAAs in a colonoscopy-based case-control study among women, but saw no association for individual or total HAAs among men [26]. We also found no apparent association for dietary HAA intake in a colonoscopy-based case-control study among Japanese-Brazilians in São Paulo, who had different dietary habits and consumed fish and meats prepared with different cooking methods to those used by Japanese in Japan [28–30]. Meanwhile, a recent meta-analysis of colorectal adenoma showed statistically significant associations for PhIP, MeIQx, and DiMeIQx [13]. However, very few studies have examined the dietary intake of MeIQ [31]. This is because MeIQ was not detected in any meat samples evaluated by Sinha and colleagues [6], and was accordingly not evaluated in studies using the CHARRED database. In our study, in contrast, MeIQ was detected mostly from cooked fish, as shown in Table 1, which - together with the generally higher consumption of fish than animal meat among Japanese - allowed us to examine the association of MelQ intake with the risk of colorectal adenoma. Although MeIQ is less abundant in foods than PhIP, it is a more potent mutagen [3], which might partly explain the observed association. Nevertheless, the reason for the discrepant findings in men and women is unknown.

Few studies have investigated the association with stomach cancer. We found no association between dietary HAA intake and risk of stomach cancer based on a hospital-based case-control study in Nagano [25]. To our knowledge, the first report from a large prospective cohort study in the US observed a significant positive association between DiMeIQx intake and the risk of gastric cardia cancer [8].

For prostate cancer, Koda et al. found that higher intake of individual and total HAAs was significantly associated with increased risk of prostate cancer in a hospital-based case-control study in Tokyo [27]. However, a recent meta-analysis of five prospective cohort studies showed no association of PhIP, MeIQx, or DiMeIQx with the risk of either total or advanced prostate cancer [10]. Further accumulation of evidence in Japanese is therefore required, including prospective cohort studies.

Individual genetic predisposition may influence the degree of HAA metabolism and thus partly contribute to the development of cancer. Among four case-control studies in Japan, one study each for colorectal, stomach, and prostate cancer investigated whether *NAT2* acetylation genotype and *CYP1A1* and *CYP1A2* genotype modify the association between dietary HAA intake and risk of cancer [24, 25, 27]. A case-control study of colorectal adenoma tested for interaction between HAA intake and *NAT2* acetylation genotype on the risk of colorectal adenoma [26]. Although studies for colorectal and stomach cancer and colorectal adenoma observed no effect modification by *NAT2* acetylation genotype or *CYP1A1* and *CYP1A2* genotype [24–26], statistically significant interactions were found in a case-control study of prostate cancer [27]. Of interest, higher ORs were found among men with the *NAT2* slow acetylation genotype or *CYP1A2* CA + AA genotype in the highest tertile category of total HAA intake [27]. These findings from gene-environment interactions may aid our understanding of the biological mechanisms underlying an association between dietary HAA intake and the risk of prostate cancer.

## Conclusions

We developed a practical method for assessing dietary HAA intake using an FFQ among Japanese and evaluated its validity for use in epidemiological studies by comparison with PhIP levels in human hair. Evidence from four case-control studies in Japan may contribute to the assessment of carcinogenicity in humans. However, findings from epidemiological studies are limited and inconsistent, due to the difficulty of assessing exposure levels of HAAs. In particular, to overcome the limitations of assessing HAA exposure based on an FFQ, there is critical need to develop and validate stable and long-term biomarkers of HAA exposure for epidemiological use. As mentioned above, PhIP level in human hair reflects PhIP exposure over a period of weeks to months, and is a promising biomarker. Moreover, various metabolites of HAAs, such as DNA, hemoglobin, and serum albumin adducts, have been investigated [4]. Considering that the formation of DNA adducts is a crucial process in induced carcinogenesis, DNA adducts might also be exposure biomarkers. Although used in only a few epidemiological studies so far, PhIP-DNA adducts were shown to be not significantly but rather only modestly associated with an increased risk of prostate cancer among 534 nested case-control pairs within a historical cohort of men with a benign prostate specimen in the US [32]. Nevertheless, we welcome further evidence from prospective cohort studies among Japanese based on dietary HAA intake estimated by FFQ. In addition, studies using different assessment methods for HAA exposure, such as biomarkers, would be particularly valuable.

## Data Availability

Not applicable.

## Abbreviations

CI: Confidence interval
CYP: Cytochrome P-450
4,8-DiMeIQx: 2-amino-3,4,8-trimethylimidazo [4,5-*f*]quinoxaline
7,8-DiMeIQx: 2-amino-3,7,8-trimethylimidazo [4,5-*f*]quinoxaline
FFQ: Food frequency questionnaire
HAAs: Heterocyclic aromatic amines
IARC: International Agency for Research on Cancer
IQ: 2-amino-3-methylimidazo [4,5-f]quinoline
JPHC study: Japan Public Health Center-based Prospective Study
LC-ESI/MS/MS: Liquid chromatography-electrospray ionization/tandem mass spectrometry
LOD: Limit of detection
LOQ: Limit of quantification
MeIQ: 2-amino-3,4-dimethylimidazo [4,5-*f*]quinoline
MeIQx: 2-amino-3,8-dimethylimidazo [4,5-*f*]quinoxaline
NAT1: *N*-acetyltransferase 1
NAT2: *N*-acetyltransferase 2
OR: Odds ratio
PHC: Public health center
PhIP: 2-amino-1-methyl-6-phenylimidazo [4,5-*b*]pyridine
Trp-P-1: 3-amino-1,4-dimethyl-5*H*-pyrido [4,3-*b*]indole
